# Cities and the SDGs: Realities and possibilities of local engagement in global frameworks

**DOI:** 10.1007/s13280-022-01714-2

**Published:** 2022-03-04

**Authors:** Amelia Leavesley, Alexei Trundle, Cathy Oke

**Affiliations:** Melbourne Centre for Cities, Faculty of Architecture, Building and Planning, University of Melbourne, GO1, Building 113, Melbourne, VIC 3010 Australia

**Keywords:** City actors, Engagement, Local government, SDG localisation, Transition management

## Abstract

City action is critical to achieving global visions for sustainability such as the UN’s Sustainable Development Goals (SDGs). However, SDG ‘localisation’ is complex procedure, with divergent outcomes depending on context and diverse city processes. This paper considers the operational challenges faced by city actors in taking on the SDGs, and subsequent implications for initiating local (and global) sustainability transitions. We analyse emergent approaches to SDG localisation within the Asia–Pacific, using a policy analysis framework (transition management) to assess transformation potential. We find that SDG localisation can influence urban sustainability, but effective implementation requires sufficient data, resourcing, and guidance—which are not readily, nor equally available to all city governments. City-to-city peer learning can accelerate SDG uptake, but realising the transformative ambition set out by the SDGs will require an approach to localisation that clearly demonstrates why and how any city government can and should engage with global sustainability frameworks.

## Introduction

Cities are critical drivers of global change. They produce 80% of global economic output (UNDP 2016), consume 70% of global resources and energy supply (UN-Habitat 2019), and, relatedly, account for 75% of human-induced carbon emissions (UN-Habitat 2020). In their current form cities are both unsustainable and inequitable, generating a disproportionate level of emissions and other forms of waste, whilst drawing heavily on resources from their hinterlands and beyond (Steffen et al. 2018). However, there is also increasing recognition that our current and future cities will be central to any transition to planetary sustainability (Parnell 2016; Oke et al. 2021). As the world grapples with the impacts of climate change, biodiversity loss, and other socio-economic disruptors such as the ongoing COVID-19 pandemic (Corbett and Mellouli 2017; Thwaites et al. 2020), city governments are emerging as agents of change: well-networked, community-oriented, and primed to transform the urban agenda (Parnell 2016; Satterthwaite 2016).

The term ‘city’ here refers to a geographically distinct, sub-national area with majority urban population. As cities proliferate, “there is no longer a question of whether cities are important to sustainable development”, but rather, how urban development can—and will—“affect our common future” (Parnell 2016, p. 529). It is through this normative, functional lens that we consider the complexity and dynamism of urban systems; domains that must be urgently refocused and transformed if global visions of sustainability are to be achieved (Ernst et al. 2016).

At this distinct sub-national scale, local authorities within city governments^1^—hereinafter city actors—play a significant role in decision-making, delegated to varying degrees by national legislative bodies. Increasingly, city actors are engaging directly in global diplomacy and multilateral governance. However, the extent to which this engagement is impacting local and global sustainability transitions is unclear.

In this paper, we examine city actor engagement within one such global framework for sustainability: the United Nations’ Sustainable Development Goals (SDGs). The research draws upon case studies from within the Asia–Pacific—one of the world’s most rapidly urbanising regions—to investigate emergent approaches to SDG localisation: a process of integrating these global goals into local planning procedures and calibrating them with the city scale. After defining the methodology and case material, we conduct an analysis using policy success factors from the four spheres that describe transition management—strategic, tactical, operational, and reflexive—to identify the mechanisms that influence urban sustainability transitions (Loorbach 2010; Wittmayer et al. 2018). The findings are discussed in relation to: (i) enabling factors for effective engagement with the SDGs by city actors; (ii) limitations to current localisation approaches; and (iii) mechanisms to accelerate uptake through peer networks.

## Background and theoretical framework

### Multilateralism, sustainability and cities

Throughout the twentieth century institutionalised nation state multilateralism provided the primary mechanism for addressing global problems, with varying levels of effectiveness and efficiency (Parnell 2016). Foremost amongst these multilateral institutions is the United Nations (UN), which put forward *Transforming our World: The 2030 Agenda for Sustainable Development* (hereinafter the *2030 Agenda*) as the preeminent framework for steering international development efforts. Adopted by all 193 UN Member States in 2015, the *2030 Agenda* moves beyond the socio-economic development objectives of its predecessor, the *Millennium Declaration*, by considering human development in tandem with planetary boundaries (Sachs et al. 2019b).

The *2030 Agenda* centres upon the seventeen SDGs, which in turn contain 169 targets, and 231 unique indicators. This highly ambitious suite of cross-sectoral objectives—developed through an intensive process of negotiation and consultation—can be summarised as aiming to “end poverty, fight inequality and injustice, and tackle climate change by 2030” (Servaes 2017, p. 2). The *2030 Agenda* is, however, not without its critics. Progress toward all of the seventeen SDGs at a global scale has been slow (Moyer and Hedden 2020). Six years in, “no country is on track to meeting all goals” (Sachs et al. 2019a, p. viii). Furthermore, efforts to accelerate implementation of the SDGs throughout the 2020s in a ‘Decade of Action’ have been derailed by the COVID-19 pandemic (Thwaites et al. 2020).

The inclusion of an ‘urban’ goal, SDG11, which seeks to “make cities and human settlements inclusive, safe, resilient and sustainable” (UN 2015), reflects increased global recognition of the critical role that cities have to play in achieving sustainable development. The targets within SDG11—ranging from affordable housing to disaster resilience—highlight not only the diversity of transformative actions required within cities, but also the role that local governments can play within these multilateral frameworks.

### Mapping city engagement with the SDGs

In response to the SDGs, alongside other global sustainability agendas such as the *Paris Agreement*, city actors are becoming increasingly engaged in international decision-making (Hartley 2019; Pipa and Bouchet 2020). Reflective of this is a mounting body of literature on localisation initiatives in cities (e.g., Simon et al. 2016; Valencia et al. 2019; Fox and Macleod 2021), which have grown alongside the proliferation of SDG localisation reports, known as Voluntary Local Reviews (VLRs). VLRs are modelled on the state-focussed Voluntary National Review format set out in the *2030 Agenda*. SDG reporting has consolidated around the VLR approach. More than 200 such reports have been developed, or are under development, by cities globally (NYC-OIA 2020), together with a number of regional guidelines. According to Fox and MacLeod (2021, p. 4)—who also co-authored the VLR Handbook for United Kingdom cities—these regional guidelines focus on “a four-step process of locali[s]ation initiated by central, regional or local governments: awareness, advocacy, implementation and local monitoring”. These stepwise frameworks describe a linear process of engagement designed to help guide the localisation process—which, at the time of this research, was still in the initial stages of development—and provide tools to initiate local dialogues.

Drawing on their experience localising the SDGs in Bristol, Fox and MacLeod (2021, p. 13) claim that “SDG-inspired networks are often touted as a means to cultivate peer-to-peer learning, [but] there is (as yet) little concrete evidence that this is happening”. Here, we challenge this statement by presenting findings from a city actor localisation project, the “SDGs Cities Challenge” (hereinafter the Challenge), as an example of awareness raising through a cohort of city actors as the starting point for SDG engagement (step 1 in the localisation process). We combine these with a review of VLR reports (considered as step 3 in the process: implementation) from the same region and apply a policy analysis framework from sustainability transitions research—transition management—to assess city-scale engagement with the *2030 Agenda*. Our method sits above these stepwise frameworks as a transition approach, where we investigate the complexities of moving from SDG awareness to implementation and monitoring.

We position our research as an exploration of step 1 of the localisation process—described as raising awareness to generate interest and stakeholder support for the SDGs (Fox and Macleod 2021)—by responding to necessary questions of *who* is doing the awareness raising, *how* and *why*. We combine this with a preliminary investigation into the challenges faced by cities as they transition from initial engagement to SDG implementation, and the role of peer-to-peer learning in supporting this process. In doing so we examine mechanisms that cities utilise to start engagement with the localisation process, and, secondarily, whether the study participants consider the localisation process as an opportunity for local transformation.

### Sustainability transition theory and transition management

To effectively localise the *2030 Agenda* (i.e. effectively use the SDGs to direct and transform urban planning processes), and in doing so transition our current and future cities toward sustainability, urban development must undergo radical systemic change (Ernst et al. 2016). Sustainable transition theory is an emerging field of scientific research that recognises cities as complex, dynamic systems within the broader normative context of sustainable development (Wolfram and Frantzeskaki 2016). Sustainability transitions are understood as “multi-level, multi-phase processes of structural change in societal systems” (Loorbach 2010, p. 166). Born from socio-technical systems research (Van Den Bergh et al. 2011), recent applications of this theory have explored socio-institutional interactions (Loorbach et al. 2017), including urban governance (Frantzeskaki et al. 2018).

The conceptual framework for this research, transition management, is a subset of sustainable transitions theory (Geels 2005; Loorbach 2007). Transition management provides a policy analysis framework for understanding the theoretical dynamics of transformative change (Frantzeskaki et al. 2018). It is regularly used as an operational model for transition governance, including for the management of urban sustainability transitions (Wittmayer et al. 2018). The four ‘spheres’ of transition management—strategic, tactical, operational, and reflexive (Loorbach 2010)—have been applied here to categorise city-level engagement with the SDGs and to assess transformative potential of this engagement*.* These spheres describe four different types of governance activities, which are outlined below.

Strategic elements focus on the long term and seek to influence cultural change (Wittmayer et al. 2018), including activities such as vision development, long-term goal formulation, and setting achievable targets (Loorbach 2010). Tactical elements relate to system structures, and include the institutions, regulatory frameworks and actor networks that influence how society functions. Activities within this sphere focus on programme development, stakeholder engagement and cross-sectoral policy integration (Wittmayer et al. 2018). The operational sphere includes activities that relate to project delivery (Loorbach 2010), such as resource provision, skills and knowledge, and utilising a range of policy mechanisms for implementation (Bush 2020). This level of practice focuses on individual experiments and actions that shape the way a system operates (Wittmayer et al. 2018). Reflexive elements focus on iterative adaptation, and include the monitoring, assessment and evaluation of all ongoing activities (Loorbach 2010). These elements are required to assess the effectiveness of policy implementation (Wittmayer et al. 2018).

Consistent with transition management, the methodology applied in this research is grounded in a post-positivist perspective, acknowledging the existence of multiple worldviews to support a broader critical inquiry into complex socio-cultural systems (Guba and Lincoln 1998). Post-positivism is underpinned by the assumption that plausible descriptions of reality are best achieved through empirical observation and experimentation in multiple settings (Harré 2012). Reflective of this, the research uses a mixed methods approach known as critical multiplism, which seeks to undertake inquiry in more natural settings by employing a combination of methods such as participant observation and engagement in combination with objective quantitative assessments like document reviews (Guba and Lincoln 1998). The aim of this approach is to falsify predetermined hypotheses about how the world works, which makes it useful for addressing broad issues (Shadish 1993). The research approach set out below uses varied and valid methods to gain a more comprehensive understanding of complex system dynamics.

## Materials and methods

### Overview

City engagement with the SDGs to date has primarily involved a process of policy integration, conducted with city networks and other urban actors. Many of these initial efforts have been institutionalised through the production of a standalone report or reports, setting out how these global goals interact with existing local systems of governance. These two components—(i) city-to-city networking facilitated through city actors, and (ii) associated documentation of policy integration—provide the primary sites of inquiry for this research, focused within the rapidly urbanising Asia–Pacific region.

Two SDG localisation datasets provide the materials for this research. The primary dataset was collected through a combination of participatory observation and semi-structured interviews with the participants of the Challenge. The secondary set constitutes a database of VLRs produced by cities in the Asia–Pacific. Sampling and analytical methods are outlined below. The research triangulated findings across these two actor-level and documentary components of the localisation process through the common lens of transition management, building on a tailored suite of mixed methods, integrated in analysis through thematic coding (using the programme NVIVO).

### Approach and rationale

As published records of SDG engagement, the VLRs provide a useful mechanism for understanding social organisation (Coffey 2014). However, transformative change is an evolving process and as ‘static’ documents (Marotzki et al. 2014) VLRs provide limited insight into the situational dynamics that drive local change. Therefore, their analysis has been supplemented by direct engagement with city actors; qualitative data that has enabled a deeper understanding of the underlying socio-political systems that influence a city’s approach to SDG engagement and, crucially, affect transformative capacity. This combination of methods was designed to capture broad regional commonalities as well as the intricates of local policy integration.

### Study sample

The sampling environment focused on urban areas in the Asia–Pacific region, which are home to more than half of the world’s urban population and many of the fastest growing cities (UN-DESA 2018a, b). As the most rapidly urbanising region in the world (UN-DESA 2018b), the Asia–Pacific has become a recognised hotspot for high-impact sustainable urban development (Mohan 2006).

**Fig. 1:**
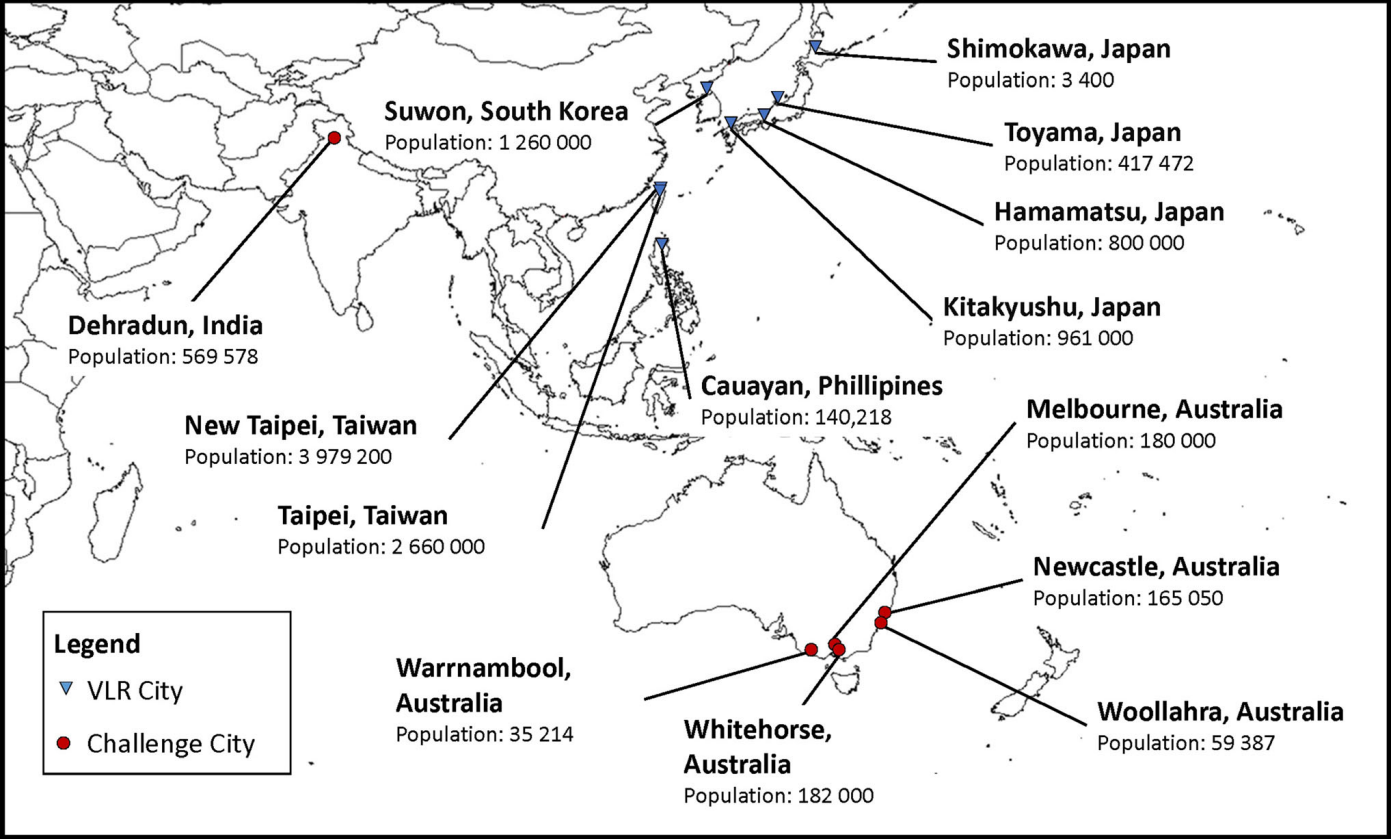
Study sample: cities in the Asia–Pacific localising the SDGs. (Source: author)

The primary data involved six semi-structured interviews with city participants from the first annual iteration of the Challenge, a cooperative research project designed to engage city actors in the localisation process by defining a set ‘challenge’ and framing implementation using targets from SDG11 (Connected Cities Lab 2020). Interview participants were recruited from the 2020 Challenge programme, which included policy officers and planning consultants from six cities in the Asia–Pacific region: Dehradun (India), Melbourne, Newcastle, Warrnambool, Whitehorse, and Woollahra (all from Australia) (shown in Fig. 1). The interview questions were designed to investigate considerations by city actors inherent in applying the SDGs and were organised around three primary themes—(i) rationale for SDG engagement (i.e., why is your city interested in the SDGs?), (ii) important aspects and barriers involved in localisation (i.e., what are some of the difficulties / opportunities for embedding the SDGs into your city?), and (iii) how to enable transformative change in line with the SDG framework (i.e., what would transformative change look like in your city?)—with supplementary questions to prompt discussion.

The primary data analysis was supplemented by a document review in which all publicly accessible, city-scale VLRs from the Asia–Pacific region, available in English were analysed (current to July 2020). This included eight VLR documents in total, published by the following cities: Shimokawa, Kitakyushu, Hamamatsu and Toyama in Japan, Taipei and New Taipei in Taiwan, Cauayan in the Philippines, and Suwon in South Korea (also shown in Fig. 1).

### Analytical methods

Analysis integrated approaches to SDG localisation in cities with transitions research, using transition management’s four spheres of governance activity—strategic, tactical, operational, and reflexive—to understand and analyse how cities undergo transformative change (Loorbach 2010; Frantzeskaki et al. 2018). This approach is known as *directed content analysis*, which uses existing theoretical knowledge to focus the analysis (Hsieh and Shannon 2005). The analytical framework (Table 1) provided a standardised approach to enable qualitative analysis of SDG localisation approaches from different cities.

**Table 1:** Success factors for managing sustainable transitions, adapted from Loorbach (2010) and Frantzeskaki et al. (2012)

Transition management spheres	Type of change	Policy success factors/analysis elements
Strategic	Cultural	Vision and objectives, goals and targets, leadership, internal buy-in (champions)
Tactical	Structural	External buy-in (alliances and partnerships), community engagement, policy integration
Operational	Practical	Technical skills, delivery mechanisms, resources for implementation
Reflexive	Temporal	Indicator sets, monitoring and evaluation, city to city learning

Six interview transcripts and eight VLR documents were coded systematically using the transition management framework and frequently used words were extracted and examined (Cope 2005). Interviewees were anonymised using the code *SP* to denote *Study Participant* and were differentiated using random number allocation (e.g., SP1). The results were quantified to identify regional commonalities (Bryman 2012) and the content was analysed using the transitions theory literature to understand the mechanisms and success factors for transformative change enabled by city engagement with the SDGs. Interview analysis focused on the practices (values, identities, norms) and institutional arrangements (activities, rules, regulations) that drive the *localisation process* and underpin a city’s interest and capacity to engage with the SDGs (Frantzeskaki et al. 2012). VLR analysis identified common themes in *localisation content* for cities in the Asia–Pacific (Vogel and Henstra 2015) and assessed transformative potential by benchmarking these approaches against the success factors for transition management.

## Results

This section presents an overview of the findings from the analysis of the Challenge process and VLR content. Analysis established the degree to which each city addressed the policy success factors, which were assessed according to: mention of the element (verbalised, written, or not), initiative progress (implemented, or not), and the level of reporting (detailed, or not). Detailed and specific reporting on policy intent—or more reliable but less frequently reported policy outcomes—are assumed to be the greatest measure of policy success. Enabling factors, that would inhibit or accelerate policy success, were identifiable from the qualitative interview data (only)—indicative of the limitations of the VLR desktop review. A policy success scale was developed as a comparative tool to demonstrate regional variation in SDG engagement, based on the available interview and document data, and frames transformative potential in relation to SDG implementation.

The results presented below show that both approaches rely heavily on strategic elements to spearhead city-level engagement with the SDGs, whilst tactical elements were considered important, but secondary mechanisms. Operational and reflexive elements received less attention, though they were considered “key entities” (SP3) for SDG localisation. The analysis is organised sequentially to address policy elements within the four spheres of transition management—strategic, tactical, operational, and reflexive—and outlined below.

### Addressing elements of policy success within the challenge process

The interview data provided important grounding for the analysis; looking at initial stages of the localisation process and providing deeper insight into what the SDGs, and indeed transformative change, can mean to city actors. The Challenge participants (and interview subjects) were each asked to identify a set ‘challenge’ for their city and a related target within SDG11 (outlined in Table 2) as part of the Challenge programme. The diversity of approaches to localisation (the ‘challenges’) are immediately evident and evolved even though all cities were given the same resources, directed to frame their localisation challenge using the same goal (SDG11), and encouraged to participate in the same collective learning process. The results from the interview analysis, mapped against transition management’s four spheres, are described below (Table 2).

**Table 2:** Specific localisation ‘challenges’ identified by participants as part of the SDGs Cities Challenge programme (sourced from Connected Cities Lab 2020). Participants were asked to select a local project and relate it to one or more targets within SDG11

City	SDG	City Challenge
Dehradun	11.2	Our proposal calls for a children friendly mobility plan for the city, with emphasis on providing access to safe and affordable mobility systems in their journey from home to school
Melbourne	11.7	The City of Melbourne’s challenge is using or modifying SDG targets and indicators to track and understand how well they are adapting the city to climate change impacts
Newcastle	11	Our challenge is to integrate SDG11 into our Indicator Framework
Warrnambool	11	…to find ways to effectively measure, report and track the impact of climate change and adaptation programmes in urban development within the city
Whitehorse	11.1, 11.6	…to develop a robust sustainable procurement process that measures the whole life-cycle sustainability of procured goods and services and incorporates an effective assessment and monitoring system
Woollahra	11.6	…to develop a framework to set community resource use and emissions reductions targets and to develop a strategy and system to monitor, track and report on our progress towards these targets

### Addressing elements of policy success within the challenge process

#### Strategic elements

Focused on long-term actions and the whole system (Loorbach 2010), the strategic elements from the interview analysis were concerned with changes in the dominant culture of city administration. Organisational buy-in was the stand-out success factor, and five out of six interview participants identified that they would struggle to achieve significant change without widespread internal support (*SP1,2,4,5,6*).

Creating ownership of the goals required both top-down and bottom-up approaches (*SPs 1 and 2*). High-level support and coordination provided an avenue for structural changes within the organisation: “leadership buy-in is really important…if it's made a priority, and we have to report against it” (*SP4*). On the other hand, half of the participants identified education around and familiarity with the SDGs as a barrier to uptake across other teams, outside of those who specialise in sustainability (*SP2,3,4*). All city actors who participated in the interviews were policy officers (except one, an external planning consultant) and came from a sustainability or community planning team within the organisation. More than half of the interviewees positioned themselves as champions (*SP2,4,5,6*), and sought to build momentum by demonstrating to other teams “the practical application that will come through the localisation” (*SP4*). Key to this was the use of consistent and coherent narratives, to encapsulate a clear vision and translate the SDGs into local language (*SP1,2,3,4 5,6*).

Two interviewees also identified the importance of linking the SDGs to their city’s visionary future. The organisational vision set the precedent for action and built credibility around the intent of localisation: “this aligns with our city’s vision, hence it's very important” (*SP1*).

#### Tactical elements

Elements within this sphere concentrate on changing system structures and building a shared agenda (Loorbach 2010). The tactical activities in this case sought to direct change through strategic engagement: setting priorities and seeking support from stakeholders. Both these activities were considered important, but approaches differed between the study participants.

Half of the participants believed that aligning Council priorities with SDG outcomes was the “real first step” (*SP6*) to changing system structures (*SP1,4,6*). Four interviewees mentioned the importance of project-based approaches to ‘seed’ policy innovation (van Buuren and Loorbach 2009), using pilot projects to integrate SDG principles into planning. These small-scale projects focused on producing tangible, shareable outcomes and provided city actors with the “impetus to just go ahead and do it” (*SP3*).

All participants mentioned building actor networks to support implementation efforts. Key stakeholder groups included industry, academia, non-government organisations and other local and national government agencies. Regional (or national) government bodies were perceived as “the biggest stakeholder” (*SP5*), required to support financial resourcing (*SP3,5*) or set strategic priorities (*SP4,5*). Intergovernmental collaboration was a crucial issue for cities to navigate (Hartley 2019) and higher levels of government could have considerable (*SP1,2,4,5,6*) and sometimes decisive (*SP3*), influence local policy trajectories. “Certainly, all levels of government need to be on board” (*SP1*), however most (five out of six) participants indicated that they could progress despite resistance from above.

Most participants viewed the private and academic sector as useful groups to engage for advice (*SP1,2,3,*6) and/or support for implementation (*SP3,4*,5,*6*), though this was seen as a supplementary process to “keep the momentum [going]” (*SP5*).

Community engagement was considered an important procedural process, designed to frame (*SP1,5*,6) or support (*SP3,2,4*) rather than lead implementation efforts. However, the importance of community values was often mentioned in the context of shaping Council priorities, as a “hand in hand kind of thing” (*SP1*). This indicated that community groups do influence the agenda setting process, “where the community thinks Council’s role is… that’s really important” (*SP6*). However, the ways in which community groups influenced a city government’s efforts to localise the SDGs, beyond strategic prioritisation, remained unclear.

#### Operational elements

This sphere focuses on the operational elements that support policy implementation (Loorbach 2010), which, in this context, referred to activities that directly contribute to—or detract from—SDG localisation. Inadequate resourcing, including financial and non-financial provisions, was highlighted as a limiting factor (*SP2, 3, 4,* 5) or at least an influencing factor (*SP1*) that affected a city government’s capacity to effectively localise the SDGs. Limited access to data was also identified as a barrier to benchmarking processes (*SP3*) and gaining traction on the ground: “there is a large data gap…and without data you’re just another person with an opinion” (*SP3*).

In addition to resource provision, access to technical skills and knowledge were identified by all participants as necessary tools to support engagement with the SDG framework. “Taking on the SDGs is a really big kettle of fish” (*SP1*). Four out of six of the interviewees (*SP1,4,5,6*) indicated that that knowing where to start was the most difficult aspect to implementation, due to the complexity of the framework and the need for locally relevant data: “how, as an organisation, do we implement or report or embed the SDGs?” (*SP5*). Many found the Challenge process useful, “the focus on SDG11 helped us to understand all the layers that are under 11, and then replicate that across all the SDGs” (*SP5*). Seeking support from ‘experts’ in academia and industry was pivotal to the learning process, as it provided “reassurance in terms of where we are heading” (*SP1*).

#### Reflexive elements

Focusing on system surveillance and adaptive learning, elements within this sphere are designed to reflexively monitor, evaluate and adjust activities to better enable a smooth sustainable transition (Loorbach 2010). Efforts to monitor and evaluate progress toward the SDGs was considered important to almost all the study participants “monitoring and evaluation is a very key entity … [this process] makes it easier for you to change the course if there is a necessity” (*SP3*). Localised indicators were considered important for benchmarking efforts and tracking progress toward the goals, and some participants suggested they would struggle to gain traction due to poor, or irrelevant, data and procedural complexities (*SP3,5*).

Reflexive elements also included processes of learning, and all participants indicated that city-to-city networks were important for sharing lessons learned and inspiring ongoing improvement. Peer networks fostered a culture of collective problem solving that enabled newcomers to “lean on Councils that have done work in this space” (*SP6*). Most participants mentioned that working with other cities provided enormous value to them, as they navigated the journey toward SDG localisation. City-to-city peer learning, facilitated by the common language of the SDGs, was also described as a useful platform elevate local problems—and seek solutions—in an international context (*SP1,4,5*). For some participants, access to this city-network was the incentive to start engaging with the SDGs: “if there is any doubt, we know who to reach for when we start implementing the framework … you just have to start the transition, to the SDGs” (*SP5*).

### Addressing elements of policy success within the VLR content

The VLR analysis complimented the interview data by investigating more formalised approaches to SDG implementation. The analysis included eight Asian-Pacific cities, each with varying local characteristics and approaches to localising the SDGs. The content from each VLR was mapped against the success factors from transition management, with varied results. In general, the cities shared a common approach to monitoring progress toward SDG implementation, but each VLR had unique characteristics that demonstrate the local government’s own understanding of, and interests in relation to, the *2030 Agenda*. Success factors extracted from the four spheres of transition management bring attention to elements within each VLR that could influence policy success. The results from this analysis are summarised in Table 3, and then explained sequentially with reference to the four transition management spheres.

**Table 3:** VLR analysis, exploring synergies between the content involved with SDG localisation and the success factors for transition management

City	VLR Year	# pages	Strategic			Tactical		Operational		Reflexive	
			Vision, objectives	Goals, targets	Mayoral endorsement	Supporting strategies	Alliances, engagement	Dedicated resources	Delivery mechanisms	Monitoring, evaluation	City-to-city learning
Cauayan	2017	7	x	x^	–	–	x	–	x	–	–
Kitakyushu^+^	2018	58	xx	xx	x	xx	xx	x	xx	x	x
Shimokawa^+^	2018	60	xx	xx	x	x	xx	x	x	xx	x
Toyama^+^	2018	56	xx*	xx	x	xx	x	x	x	–	x
Hamamatsu^+^	2019	48	xx*	x^	x	xx	xx	–	x	x	x
Suwon^+^	2018	35	x	xx	x	x	x	x	xx	x	x
New Taipei	2019	131	xx	x	x	x	x	x	x	x	x
Taipei	2019	80	xx	xx	x	x	x	x	xx	–	x

- Not stated, x Included but with little detail, xx Detailed coverage, ^+^Document co-created with partner organisation, *Vision beyond 2030, ^No targets specified

Each city had different strengths and weaknesses in their policy approach to SDG implementation, demonstrated by the level information provided for the policy success factors (listed in Table 2). If a success factor was described with a high-level, or partial, overview of specific content, it was categorised as ‘included but with little detail’. For example, information on dedicated resources from Kitakyushu’s VLR was considered a high-level description only, as reflected in the statement that “plans are also in place in Kitakyushu to mobilize funds and human resources in the future” (Ota et al. 2018, p. 35). Conversely, VLRs that provided significant information in relation to a policy success factor and how it was linked to SDG localisation, like the comprehensively integrated vision and objectives from the Japanese VLRs, were classified as ‘detailed coverage’ in the analysis.

### Strategic elements

Strategic aspects were the most comprehensively covered by the VLRs, compared with the other spheres. This is likely because city governments had to first clarify how the global goals fit strategically into local context, which is reflective of the nascent stage in the localisation process that most cities are at in the region. High-level support was a key strategic element; all but one VLR included a letter of mayoral endorsement. Most documents were led by a vision and objectives that focused on attaining goals by 2030 (and some beyond that), but local interpretation and application of the SDGs varied with context. For example, Hamamatsu’s vision, to become “a creative city built on civil collaboration, shining into the future” was supported by three “pillars of actions” which related to the economic, social and environmental dimensions of sustainable development (IGES & City of Hamamatsu 2019, p. 3). Conversely, Shimokawa’s vision incorporated the SDG principle of inclusion and proposed to create “a sustainable town that is strong and resilient, where people can live happily, and no one is left behind” (Kataoka et al. 2018, p. 4).

### Tactical elements

Many VLRs suggested the importance of supporting strategies and actor networks but provided little information about how this impacted implementation. The tactical elements were most comprehensively covered by the Japanese VLRs, which shared structural similarities as they were all co-created with the Japanese Institute for Global Environmental Strategies (IGES). These VLRs included a governance framework that featured the SDGs and described relevant local policies and institutional arrangements. The influencing role of national governments was noted in more than half of the VLRs, however the level of impact on local implementation was unclear. Many of the VLRs touched on the importance of engaging with different stakeholder groups, such as academia, private business, non-governmental organisations and community groups in the context of ‘leaving no-one behind’ (UN 2015). However, information surrounding the impact of engagement on localisation efforts was limited.

### Operational elements

Operational coverage was mixed across the VLRs. For example, Suwon outlined several case studies to demonstrate existing policy mechanisms employed by the local government,^2^ and yet, only mentioned that the city would need both “administrative and financial support” for implementation (City of Suwon 2018, p. 18). Conversely, Taipei provided a detailed summary of implementation activities and mentioned a plan to gather resources (City of Taipei 2019). Within most of the other VLRs, the supporting structures for implementation (means and methods for project delivery) focused on the policy environment and governance frameworks (tactical elements). Most VLRs included some case studies to demonstrate localisation efforts, and few mentioned any dedicated resources to support delivery.

### Reflexive elements

The reflective elements were the weakest of the elements analysed. Efforts to monitor and evaluate SDG implementation were poorly reported, or simply not mentioned. The exception was Shimokawa who, possibly due to their small size and access to applicable data, were able to report on several indicators within all the SDGs. Interestingly, city-to-city learning was noted by almost all the VLRs, but there was little detail provided about what this involved and how it would influence SDG implementation. This supported the premise evidenced in the literature that city-to-city learning is a valuable outcome of localisation (Webb et al. 2018); an area that was able to be better explored in the interview analysis (above).

## Discussion

The results set out in the previous section have been synthesised, integrating findings from the semi-structured interview coding and the VLR review to more fully examine how—and why—city actors are engaging with the SDGs. It is this synthesis that forms the basis of the discussion here, coupling the ‘outward facing’, formal records of SDG deployment with the internal and informal reflections of city actors engaged at a more formative, early stage of the localisation process. In doing so we respond directly to the two core aims of this research, being to identify: (i) what aspects of transition management are being actively deployed by cities looking to localise the *2030 Agenda*, and (ii) how localised SDG engagement could drive broader social transformations to a sustainable and equitable global future, as envisaged through the SDGs.

**Fig. 2:**
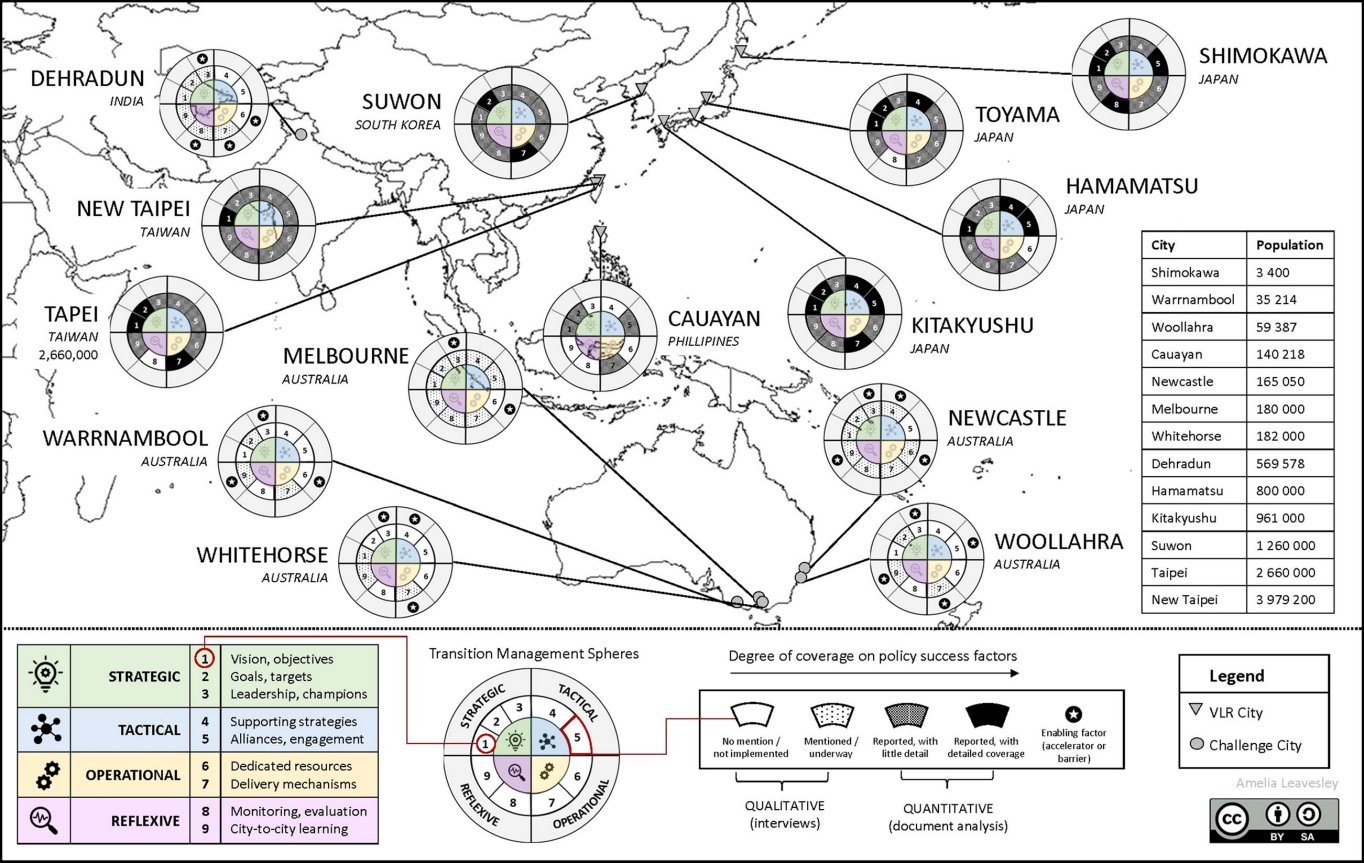
Overview image of analysis outcomes across fourteen cities within the Asia–Pacific region. (Source: author)

A summary of this synthesis is provided in Fig. 2, within which each city is represented in relation to their engagement with both transition management spheres (strategic, tactical, operational, and reflexive), and the associated policy success factors within each sphere. The discussion below identifies (i) enabling factors for effective engagement with the SDGs by city actors, (ii) limitations to current localisation approaches, and (iii) mechanisms to accelerate widespread uptake of the *2030 Agenda* by city governments in the Asia–Pacific.

### Assessing transformative potential

Widespread and effective localisation of the SDGs is challenging because of the heterogeneity of the urban form and its associated modes of local governance. There are a multiplicity of city typologies, which vary based on specific contextual attributes such as population density, geographic orientation, and the socio-economic landscape (Ju and Rhee 2020). Urban governance models are equally diverse: cities apply different regulatory systems and operate with varying degrees of sub-national autonomy, even within single nation states (Davoudi and Sturzaker 2017).

Reflective of this, each city in this study approached localisation from a different angle. As emergent SDG reports, the VLRs focused primarily on structural elements—reorganising priorities, linking the goals to local policy frameworks, and setting up institutional structures for implementation. Some VLRs addressed all seventeen goals but provided little detail about tracking specific targets and indicators (e.g., Cauayan, Toyama and New Taipei), whilst others covered a handful of relevant goals or targets in detail but did not address the overarching SDG framework (e.g., Hamamatsu, Kitakyushu, and Suwon). This diversity in approaches from cities in the Asia–Pacific is consistent with findings from other regions (Ciambra et al. 2020), where selective approaches to implementation reflected differing city actor interests (Pipa 2019).

Unlike the breadth of approaches covered in the VLRs, the Challenge project was designed to accelerate localisation of urban goals and targets, with a focus on specific enablers, such as collaborative learning and knowledge networks (Connected Cities Lab 2020). The 2020 class was conceived as ‘deep dive’ to help cities get started. The programme focused on couching SDG11 in SDG17 to build knowledge, skills, and, most importantly, partnerships to support engagement (Connected Cities Lab 2020). Participants were encouraged to frame implementation using targets from SDG11 and worked with a cohort of other cities and key stakeholders to accelerate localisation. Whilst the SDG11 framing did limit the scope of implementation, the process was designed to build capacity and skills to support meaningful engagement with the broader framework (step 1 in the localisation process: awareness raising). These limitations did not seem to hamper the diversity of approaches, as each city tailored their ‘localisation challenge’ to fit with the characteristics of their own context.

Analysis showed that the VLRs touched on most elements within the transition management framework, albeit sparingly, suggesting that there is some potential for transformative change within this emergent reporting framework. However, these documents provided little insight into underlying institutional dynamics and/or local capacity to manage ongoing systemic change, both of which can influence transformative capacity (Loorbach 2010). This is a recognised data limitation and more research into the VLR production process may provide better understanding of these processes. The interview analysis provided contextual insight into the initial stages of city-level engagement with the SDGs, and highlighted the importance of high-level support, organisational buy-in and consistent narratives for getting started. However, the analysis also indicated that access to resources, data, skills, and knowledge (Bush 2020)—as well as competing priorities and short political cycles (Klopp and Petretta 2017)—would influence a city government’s capacity to make progress toward the SDGs, beyond the initial stages of engagement (awareness raising and advocacy). This is consistent with other research that found lack of comparable methods and data variability hindered successful implementation of urban goals (Boyer et al. 2015).

Assessed through the lens of transition management, effective SDG localisation—and the subsequent transition toward urban sustainability—relied on a city government’s capacity to address all four spheres of the transition framework (strategic, tactical, operational, and reflexive) (Frantzeskaki et al. 2012). Each city had different strengths and weaknesses in their policy approach to SDG localisation, and comparability was low even though they were arguably undergoing the same process of policy integration. Whilst a flexible approach is strongly supported for widespread application (Pipa 2019) and “participation with any means is essential” (Ciambra et al. 2020, p. 34), city governments require access to comparable and locally relevant data, as well as concrete reporting mechanisms, to effectively track progress toward the *2030 Agenda*. Without these mechanisms, city governments will struggle to measure the impact of localising the SDGs.

### SDG engagement driving local sustainability outcomes

The results from this study indicate that engaging with the SDGs can influence sustainable development outcomes on a local level. Despite resource limitations, local governments are already primed to implement sustainable development principles into planning (Hartley 2019). Local policy structures are set up to respond to urban development issues, such as climate change and public health, the outcomes of which align with many SDG targets (Lucci and Lynch 2016). Several study participants indicated that embedding the goals into local context would be a process of “re-jigging” (*SP6*) plans and processes to align with the SDG framework. The applicability of the SDG framework was also evident in the VLRs, as many city governments deployed the SDGs by restructuring strategic priorities and aligning existing initiatives with specific goals.

Local engagement with the SDGs also proved a useful mechanism for embedding sustainability principles into urban planning. Urban sustainability strategies typically require articulation of the problem, vision development, and construction of a plan to achieve the vision (Etzion 2018). Integrating the SDGs into high-level city governance documents, such as a Community Strategic Plan (used by some local governments to direct strategic priorities over the political term), sets a normative agenda within the organisation and orients policy toward achieving sustainability outcomes (Wittmayer et al. 2018)*.* Policy integration also breaks down organisational silos, linking policy makers from across different domains and building shared ‘ownership’ of the SDGs (Bush 2020). This process of organisational buy-in is enhanced through narratives that encapsulate a shared vision (Bush 2020) and concrete methods to measure and evaluate progress (SDSN 2015). By embedding the SDGs into corporate reporting systems, city governments can monitor implementation activities and use localised indicators to assess progress toward the goals. Customising the SDGs to fit local context is an useful process for promoting internal coordination, managing performance and orienting urban planning toward global sustainability outcomes (Pipa 2019).

Analysis has highlighted elements which contribute to—or detract from—a city government’s ability to effectively localise the SDGs. Local integration of the SDG framework could be considered a process of structural and cultural change; one that involves shifting institutional practices and procedures toward a new mode of operation (Loorbach 2010). We argue that this process of cultural change relies upon four key processes: (i) developing consistent narratives to encapsulate a clear vision and increase awareness (Luederitz et al. 2017), (ii) engaging city actors (beyond the core sustainability and/or community planning teams who typically initiate engagement) through the articulation of shared values (Bush 2020), (iii) securing high-level support and coordination to align strategic priorities (Webb et al. 2018), and (iv) fostering processes of co-creation (de Haan and Rotmans 2018).

Effective implementation of the SDGs relies on the reorientation of a city government’s strategic priorities, organisational values, and regulatory activities toward sustainability outcomes (Wittmayer et al. 2018). As such, commitment to and leadership from mayors, or other elected officials, is central to transformative localisation of the SDGs. This kind of high-level support has also been shown to accelerate implementation efforts in other regions, such as Europe (Finnveden and Gunnarsson-Östling 2016), Africa (Sharma and Vora 2019) and North America (Krellenberg et al. 2019).

Whilst strong leadership can accelerate localisation efforts (Frantzeskaki et al. 2018), institutionalisation of the SDGs underpins effective long-term implementation (Pipa 2019). With SDG localisation having only emerged in recent years, it is unclear whether engagement with the *2030 Agenda* will be sustained across changes in leadership at both the political level and internally within city organisations. These considerations are critical in achieving long-term and technically detailed transitions such as those set out in the SDGs: other studies have found that policy implementation can diverge from high-level goals and objectives (Raynor et al. 2018), especially if it is hampered by lack of resources, limited skills and knowledge, and/or inadequate reporting mechanisms (Bush 2020). However, more research is needed to better understand how the SDG localisation process is sustained over time.

### Limits to (vertical) integration

To address the country-level focus of the *2030 Agenda,* city and other local actors must adapt the SDG framework for application at sub-national scales, a process referred to here as ‘localisation’ (Ortiz-Moya et al. 2020; Pipa and Bouchet 2020). Local implementation of the SDGs underpins the success of the *2030 Agenda*, but our findings highlight that the process of localisation—manifesting in the production of a VLR—is resource intensive, technically complex, and can result in a divergence of outcomes and indicators across cities. Concurrently, whilst SDG engagement by city actors can influence urban sustainability outcomes, effective localisation relies on the ability of those actors to integrate the SDGs into planning (de Haan and Rotmans 2018) and consolidate efforts toward their achievement (Klopp and Petretta 2017).

SDG localisation processes often begin with awareness raising and advocacy activities, but are sustained by ongoing, “embedded advocacy” led by passionate and informed individuals (Fox and Macleod 2021). The Challenge cities joined with an existing sustainability policy, which speaks to the importance of policy mandate to initiate SDG engagement. Additionally, the background and role of the study participants (all but one were policy officers with sustainability or planning backgrounds) is a useful observation, if not particularly surprising in relation to who is engaging (i.e., engagement starts with those who have prior knowledge / direct interest in local planning and sustainability). However, further research is required to assess the impact of ongoing commitment by these individuals and partners on attempts to localise the SDGs.

Various guidance documents are available to support the VLR process and encourage city actors to start implementing the SDGs, though the range of recommended approaches varies considerably. This abundance of localisation options is a challenge in and of itself. Without a standardised or official process, city actors have been found to doubt whether their own approach to localisation is ‘right’ or ‘valid’ (Ciambra et al. 2020) and question the legitimacy of changing an international framework (Klopp and Petretta 2017; Valencia 2019). This was consistent with the research findings here: at least half of the interview participants indicated the importance of process validation for overcoming the “scary prospect” (*SP1*) of adapting UN-set targets and indicators to suit local settings.

Regionalised guidelines, such as an Asian-Pacific VLR method (ESCAP 2020), and processes that foster city-to-city learning, such as the Challenge (Connected Cities Lab 2020), can inspire confidence and help to overcome these obstacles through shared socio-political identities, comparable data and knowledge exchange. However, city governments have limited capacity and actors may struggle to gain traction without access to a clear guiding framework, robust datasets, and/or adequate resourcing (Klopp and Petretta 2017; Ortiz-Moya et al. 2020). Effective localisation is limited by these procedural complexities, as city actors must navigate the vertical tensions that exist between locally unique challenges, regionalised guidelines, and nationally focused goals, to find a method that works for their city.

### Limits to (horizontal) exchange and replication

There is potential for city actors to become agents of change and accelerate the global transition toward sustainability from the ground-up (Ortiz-Moya et al. 2020; Pipa and Bouchet 2020). However, as we have demonstrated, integrating a broad global agenda into local context is a complex and challenging process (Klopp and Petretta 2017; Hartley 2019). Not only is the process of local (vertical) integration of the SDGs faced with procedural complexities, but widespread (horizontal) uptake by cities globally is difficult because the urban form is not homogenous (Castán Broto et al. 2019). Cities have diverse typologies (Davoudi and Sturzaker 2017), regulatory systems (Castán Broto et al. 2019), and domestic governance structures (Stafford-Smith et al. 2017) which means there is no ‘one size fits all’ approach to localisation. Compounding these challenges is the very real (and problematic) assumption that all (or even most) city governments can respond to global challenges. Generating transformative change at the local scale is proving difficult due to the inherent contextual variation (Davoudi and Sturzaker 2017) and very different institutional capacities that exist between cities (Simon et al. 2016). Not all cities were created equal, and many city actors will struggle to start engaging with, let alone implement the SDGs without access to adequate support and/or guidance.

To demonstrate this point, the Challenge initially included nine participant cities, though only six were interviewed. City actors from two Least Developing Countries (LDCs), Honiara (Solomon Islands) and Port Vila (Vanuatu^3^), did not participate in webinar activities due to resource constraints exacerbated by the COVID-19 pandemic. Additionally, Davao City of the Philippines was hit by a typhoon during the Challenge and, as such, had to withdraw from the final stages of the programme. Whilst these city actors did start engaging with the SDG framework, their capacity to integrate and achieve the goals was disrupted by turbulent events (Ansell et al. 2020). The non-participation of LDCs is also reflective of broader issues of scaling SDG localisation horizontally to secondary city contexts (McEvoy et al. 2014).

Secondary cities in developing countries make up the bulk of the global urban population, and projected growth (Cohen 2006). As such, the involvement of these urban governments will be critical if SDG localisation is to be effective at achieving global outcomes (Parnell et al. 2007; Jackson 2016). Crises such as a global pandemic or a natural disaster can exacerbate prevailing issues, including access to housing and sanitation; the impacts of which tend to disproportionately affect the most vulnerable communities (Barbier and Burgess 2020). Successful localisation of the *2030 Agenda* will require an integrated and functional approach (OECD 2020), that provides *all* city governments with the ability to respond (Ciambra et al. 2020).

### Accelerating uptake through peer networks

Alongside the benefits of local integration, the SDG framework provides a useful platform for international cooperation amongst city networks. City-to-city partnerships are considered crucial for tackling persistent urban problems and for overcoming many shortcomings in the localisation process (Webb et al. 2018). As we see, many of the VLR documents analysed in this study were co-created with partner organisations (IGES or ICLEI), which is indicative of the value of external expertise, and the additional capacity provided by these external organisations who bring the comparative lessons learned from working across many cities (Valencia et al. 2019). By connecting local strategy to a global agenda, city governments can pragmatically source and share the best solutions, drawing on transnational networks to overcome the challenges they face (Graute 2016). Urban heterogeneity provides challenges for comparability (Ju and Rhee 2020), but the universal goals are recognised as important signposts for directing collective action to address urban challenges: “the journey is not going to be the same for every council, but the end goal is the same” (*SP1*). The SDGs provide a common language to organise efforts, share experiences and spur innovation (Pipa and Bouchet 2020). These bottom-up approaches can accelerate uptake of the SDGs and build momentum for local leadership and transformation for sustainable development (Hartley 2019). ‘Leave no-one behind’ is the central, transformative promise of the *2030 Agenda* and it’s SDGs (UN 2015). However, achieving local transformation at a global scale will require a commitment from *all* city governments, not just the vanguard city actors who are currently leading SDG engagement (Pipa 2019).

## Conclusions

The success of the *2030 Agenda* relies on widespread implementation of the SDGs, and local and sub-national governments’ support is key. This study sought to understand the mechanisms used by cities to start engaging with the SDG localisation process and some of the challenges involved in transitioning from initial engagement to actual implementation. In doing so, the authors considered how localisation of the SDGs could drive transformative change at the local scale, defined here as effective implementation and widespread uptake of the goals in widely diverse cities, to respond to the transformative needs of the global sustainable development agenda. We propose peer-to-peer learning between cities, and alongside urban partners, as a catalyst to accelerate local engagement with, and eventually uptake of, the SDGs.

SDG localisation is an ongoing process: cities are continuing to engage in the VLR process, and a new cohort of cities have joined the second year of the Challenge. The findings of this study support the case for localisation of the SDGs in cities as a transformative, scalable process, manifesting in the production and update of VLRs. We argue that preparing cities to engage in the localisation process (a preliminary step that is often overlooked in VLR guidelines) requires equally careful attention. In particular more research is needed to investigate the processes cities utilise to determine their purpose for engaging in VLR production; specifically asking *why* as they localise sustainable development.

## Footnotes

1 A city (or local) government refers to the political organisation responsible managing city functions, which vary by location, but can include policy setting, urban planning, and natural resource management (Rebelo, 2019). City actors are classified as local authorities or representatives who work within, or on behalf of, a city government.

2 Suwon created their VLR in partnership with ICLEI Korea, which may explain the specific SDG11 approach (different from the other Asia Pacific VLRs) and the addition of comprehensive case studies that demonstrate the operational mechanisms of their localisation approach.

3 Though Vanuatu is now considered a developing country, having graduated from the LCD category in December 2020 (UN 2020), it was classed as a LCD at the time of this analysis.
